# Metabolomic evaluation of *Euphorbia pekinensis* induced nephrotoxicity in rats

**DOI:** 10.1080/13880209.2018.1435697

**Published:** 2018-02-09

**Authors:** Zhenzhen Liu, Yan Zeng, Pengyi Hou

**Affiliations:** a Department of Medical Function, School of Medicine, Yangtze University, Jingzhou, China;; b School of Pharmacy, Shenyang Pharmaceutical University, Shenyang, China;; c Xiangyang Central Hospital, Affiliated Hospital of Hubei University of Arts and Science, Xiangyang, China;; d Chromatography and Mass Spectrometry Division, Thermo Fisher Scientific, Shanghai, China

**Keywords:** Metabolomics, serum biochemical parameters, histopathology, LC/Q-TOF-MS, nephrotoxicity-related constituents

## Abstract

**Context:***Euphorbia pekinensis* Rupr. (Euphorbiaceae) has long been used in the Orient, while its clinical use was limited due to its nephrotoxic effect.

**Objective:** The possible mechanism of nephrotoxicity of *Euphorbia pekinensis* (EPR) and its related constituents were investigated.

**Materials and methods:** Petroleum ether (PE), acetic ether (AE) and *n*-butanol (BUT) extracted sections of EPR were separately given to Wistar rats by gavage at the dose of 3 g/kg/day for 10 weeks to determine the nephrotoxic section of EPR. Then, renal metabolic profiling of EPR after oral administration of nephrotoxic section was investigated and its related constituents were identified by LC/Q-TOF-MS method.

**Results:** The average values of creatinine (CREA) in PE, AE, BUT and control groups were 76.54 ± 9.52, 54.12 ± 10.34, 51.33 ± 5.19 and 48.23 ± 6.67 μmol/L. The average values of blood urea nitrogen (BUN) in PE, AE, BUT and control groups were 15.25 ± 3.37, 8.32 ± 0.89, 9.22 ± 1.78 and 8.47 ± 1.33 mmol/L, respectively. Only kidney section of rats in PE group showed that glomeruli had cellular or fibrocellular crescents. Renal metabolic profiling showed disturbed metabolic pathways of purine, amino acid, phospholipids and sphingolipids in EPR nephrotoxicity. A total of 25 compounds [(–)-(1*S*)-15-hydroxy-18-carboxycembrene is a new compound] in PE section and 10 compounds in rat serum after administration of PE section were identified.

**Conclusions:** This is the first time that the toxic compounds of PER and action mechanism of EPR nephrotoxicity were explored to provide a new reference for studying the toxic components of Traditional Chinese Medicine (TCM).

## Introduction

Traditional Chinese Medicine (TCM) has a unique theoretical and practical approach to treat various kinds of diseases with a long history over thousands of years. In the past 20 years, the usage of TCM has become increasingly popular all over the world (Liu et al. 2009), while the spectrum of adverse reactions and side effects also occurred with TCM (Kassler et al. 1991; Steenkamp et al. 2000; Chitturi and Farrell 2008). Therefore, comprehensive characterization of the chemical ingredients in herbal medicines is very important for quality control and understanding the mechanism of action of toxic effects. However, it is not practical to isolate every toxic ingredient from herbs, and also difficult to detect and identify every toxic chemical ingredient in TCMs for further toxicological investigations.

*Euphorbia pekinensis* Rupr. (Euphorbiaceae) (EPR) is a herbal medicine widely used not only in the treatment of oedema, antivirus, scrofula and anti-inflammatory activity, but also in fluid-purging exercise, dispersing phlegm and alleviating oppilation by purgation (Mucsi et al. 2001; Corea et al. 2004). EPR has been demonstrated to have cytotoxic activity against human cells *in vitro* (Kong et al. 2002; Liang et al. 2009). In addition, EPR exhibited HIV-1 reverse transcriptase inhibiting effect, strong histamine-release inhibitory function and strong nitric oxide production inhibitory activities (Ahn et al. 2002; Wang et al. 2006). Previous chemical researche on EPR indicated that diterpenoids, triterpenoids, flavonoids and ellagitannins are the main constituents (Singla and Pathak 1990; Jassbi 2006; Shao et al. 2011), some of which showed tumour promoting, skin-irritant and proinflammatory properties (Sosath et al. 1988; Mucsi et al. 2001; Corea et al. 2004). Moreover, our previous toxicology research indicated that EPR-induced visible hepatotoxicity and nephrotoxicity resulted from an excessive dose (Hou et al. 2013).

Metabolomics, one of the ‘-omics’ technologies involving modern chemical instrumentation (NMR, GC/MS, LC/MS) and chemometrics analysis, is commonly used to identify the biochemical pattern of endogenous metabolic constituents in biological samples and provides important information for testing a physiopathological response to a toxin-, disease- or drug-induced disturbance in an endogenous metabolic network. Thus, it has been successfully applied to evaluate drug toxicity, to make disease diagnoses and to provide diagnostic and prognostic biomarkers specific for early stages of tissue damage (Brindle et al. 2002; Mortishire-Smith et al. 2004; Waters et al. 2006; Beger et al. 2010).

The development of reliable, high-resolution mass spectrometry analytical methods and technologies, such as quadruple time of flight mass spectrometry (Q-TOF MS), distinguishes isobaric ions and enhances authenticity in the identification of the targeted analytes by providing the elemental composition (Calbiani et al. 2004). Furthermore, accurate mass measurements of product ions in MS/MS mode are helpful for the analysis and elucidation of known and unknown constituents. Therefore, Q-TOF MS has become a valuable analytical technique in TCM research.

It is widely known that the toxicity of one herb mainly existed in its toxic section. Thus, the aim of this study was to (1) determine the toxic section of EPR by serum biochemical parameters and histopathology, (2) identify biomarkers and investigate the possible mechanism of EPR-induced nephrotoxicity after oral administration of the screened toxic sections of EPR extract for 10 weeks by using kidney tissue targeted metabolomics approach and (3) develop a LC/Q-TOF-MS method which can be applied to systematically characterize the potential nephrotoxic components in rat serum after oral administration of the toxic section of EPR. This study will provide a new reference for distinguishing the toxic components of TCM by using metabolomics, serum pharmacochemistry and pathological index.

## Materials and methods

### Chemical and materials

EPR was purchased from Anguo Chinese *Chemicals* Herbal Medicine Factory (Anguo, China) and authenticated by Professor Jincai Lu (School of Traditional Chinese Materia Medica, Shenyang Pharmaceutical University, Shenyang, China); hypoxanthine, niacinamide, phenylalanine, betaine and *N*,*N*-dimethylglycine were supplied by Sigma Corporation (St. Louis, MO); corilagin, brevifolin carboxylic acid, brevifolin, ellagic acid, 3,3′-di-*O*-methyl ellagic acid-4′-*O*-β-d-xylopyranoside, 3,3′-di-*O*-methyl ellagic acid-4′-*O*-β-d-glucopyranoside, 3,3′-di-*O*-methyl ellagic acid, quercetin, yuexiandajisu C, helioscopinolide E and (–)-(1*S*)-15-hydroxy-18-carboxycembrene were isolated from EPR and fully characterized based on chemical and spectroscopic analysis (UV, IR, NMR and MS) in our laboratory. The purity of each compound isolated was more than 98% determined by HPLC analysis. Methanol and acetonitrile of HPLC grade were purchased from Fisher Scientific (Pittsburgh, PA), and formic acid of HPLC grade was obtained from Concord Tech. Co. Ltd. (Tianjin, China). Distilled water was purified using a Milli-Q system (Millipore, Bedford, MA).

### Preparation of EPR samples

The dried and pulverized plant of EPR was processed as follows: 0.5 kg EPR was extracted three times by refluxing with 5 L 95% ethanol for 2 h each time and the solvent was concentrated under reduced pressure. Then, the residue was partitioned respectively by petroleum ether (PE), acetic ether (AE) and *n*-butanol (BUT) and water. The PE, AE and BUT sections were concentrated under reduced pressure. Furthermore, PE, AE and BUT extracts were redissolved with water and diluted to a volume equivalent of 0.375 g EPR per millilitre.

### Animal treatments

Thirty-two male pathogen-free Wistar rats (200–220 g) were provided by Beijing HFK Bioscience Co., Ltd. (Beijing, China). Animal care was carried out in accordance with the Guidelines for Animal Experimentation of Shenyang Pharmaceutical University (Shenyang, China) and the protocol was approved by the Animal Ethics Committee of the Institution. They were fed with a certified standard diet and tap water *ad libitum*. All rats were randomly divided into four groups (*n* = 8/group) as follows:

1. Petroleum ether group (PEG), rats were orally treated with PE section of EPR at a dose of 3 g/kg/day;
2. Acetic ether group (AEG), rats were orally treated with AE section of EPR at a dose of 3 g/kg/day;
3. *n*-Butanol group (BUTG), rats were orally treated with BUT section of EPR at a dose of 3 g/kg/day;
4. Healthy control group (HCG), rats were orally treated with the approximately same volume water.

Blood and kidney samples of rats in the four groups were collected after continuous oral administration of different EPR sections and water for 10 weeks. Blood samples were used in the serum biochemical parameters test and the nephrotoxic constituents’ investigation. Kidney samples (left) were used in the histopathology experiment and kidney samples (right) were used in the metabolomics study.

### Serum biochemical parameters and histopathology

Serum nephrotoxicity biochemical parameters were tested by using standard clinical laboratory methods and a clinical chemistry analyzer (P800, Roche, Berlin, Germany). Creatinine (CREA) and blood urea nitrogen (BUN) were determined for the evaluation of nephrotoxicity disorders.

The kidney samples (left) were fixed in 10% formalin solution for at least 24 h, then dehydrated with ethanol solution, paraffin-embedded and stained with haematoxylin–eosin for light microscope examination.

### Screening of EPR nephrotoxicity section

CREA and BUN levels of PEG samples were higher than that of other groups and only the kidney samples of PEG displayed obvious renal injury. Based on the results of serum biochemical parameters and histopathology experiments, PE section was screened as the nephrotoxic section of EPR. Thus, the samples of PEG were used in the following experiments.

### Sample preparation

Blood samples were collected from the retro-orbital venous plexus and centrifuged at 13,000 rpm for 5 min at 4 °C, and then serum was transferred to a 1.5 mL glass vial. A 1 mL serum sample was extracted with protein precipitation with 2 mL acetonitrile. After centrifugation (13,000 rpm, 5 min, 4 °C), the supernatant was transferred to another glass vial and evaporated to dryness under a gentle stream of nitrogen in a thermostatic controller at 35 °C. Then the residue was dissolved in 200 μL acetonitrile.

Kidney tissues (250 mg) were homogenized in 2 mL acetonitrile in an ice water bath. The homogenates were centrifuged (13,000 rpm, 5 min, 4 °C) and the supernatant was transferred and evaporated to dryness under a slight stream of nitrogen. The dried residue was reconstituted in 100 μL acetonitrile.

### Metabolomics study by ultra-high performance liquid chromatography tandem mass spectrometry (UHPLC–MS/MS)

UHPLC analysis was performed by ACQUITY^TM^ Ultra Performance Liquid Chromatography system (Waters, Milford, MA). The separation was achieved on a CAPCELL PAK C_18_ column (100 mm × 2.0 mm, 2 μm, Shiseido, Japan). The column and auto-sampler temperatures were maintained at 35 °C and 4 °C, respectively. The UHPLC mobile phase consisted of 0.1% formic acid in acetonitrile (solution A) and 0.1% formic acid in water (solution B). The following gradient program was used: 35% A (0–2.0 min), 35–44% A (2.0–3.0 min), 44–50% A (3.0–4.0 min), 50–80% A (4.0–7.5 min) and then back to 35% A in 2 min. The flow rate was set at 0.3 mL/min.

Micromass Quattro micro^TM^ API mass spectrometer (Waters, Milford, MA) was equipped with electrospray ionization (ESI) interface operating in positive ion mode. The optimum conditions were as follows: the capillary voltage was 3.2 kV, while the cone voltage was 30 V; the cone gas flow was 50 L/h; the source temperature was set to 120 °C and desolvation gas was 600 L/h at a temperature of 350 °C. Data profiling of positive ions *m*/*z* from 100 to 900 was recorded at a speed of 1 s/scan with 0.1 s as the inter-scan delay for the analysis. The mass was corrected with NaCsI before the study.

The collision induced dissociation (CID) experiment was carried out to get fragmentation patterns of those potential biomarkers. Mass spectra were interpreted with available biochemical databases, such as METLIN (http://metlin.scripps.edu/), KEGG (http://www.genome.jp/kegg/), HMDB (http://www.hmdb.ca/) and SciFinder (https://scifinder.cas.org/).

### Nephrotoxicity related constituents’ investigation by HPLC/Q-TOF MS

HPLC/Q-TOF-MS analytical procedures were performed on an Agilent 1200 system (Billerica, MA) coupled with a Bruker Daltonics microTOF-Q mass spectrometer. The HPLC separation was achieved on a Venusil ASB C_8_ column (150 mm × 4.6 mm, 5 μm, Bonna-Agela, Tianjin, China) and preceded by a C_18_ guard column (4.0 mm × 3.0 mm, 5 μm, Phenomenex, Torrance, CA). The mobile phase consisted of acetonitrile (solution A) and 0.1% formic acid in water (solution B). The gradient elution condition was optimized as follows: linear gradient from 5% to 15% A (0–5 min), 15–30% A (5–18 min), 30–65% A (18–24 min), 65–95% A (24–34 min), 95% A (34–41 min) and then back to 5% A in 2 min. The solvent flow rate was 0.8 mL/min. The column temperature was maintained at 35 °C.

The Q-TOF MS was operated in both positive and negative ion modes with an ESI source. The optimized ionization conditions were as follows: capillary voltage was 4.5 kV (ESI^+^) and 3.8 kV (ESI^−^). The nebulizer pressure was maintained at 1.2 bar. Nitrogen was used as the desolation and nebulizing gas at 180 °C by gas flow of 8.0 L/min. The full scan range was set at *m*/*z* 100–1000. Formic sodium was used for mass correction.

## Results

### Screening of EPR nephrotoxicity section

In our previous study, rats were found to have renal injury after oral administration of EPR (Hou et al. 2013), but the section contributing mostly to the toxic effect was still remained unknown. Based on the results of our previous study, we tried to screen the exact nephrotoxicity section of EPR by serum biochemical parameters and histopathology experiments.

### Serum biochemical parameters

**Figure 1.**
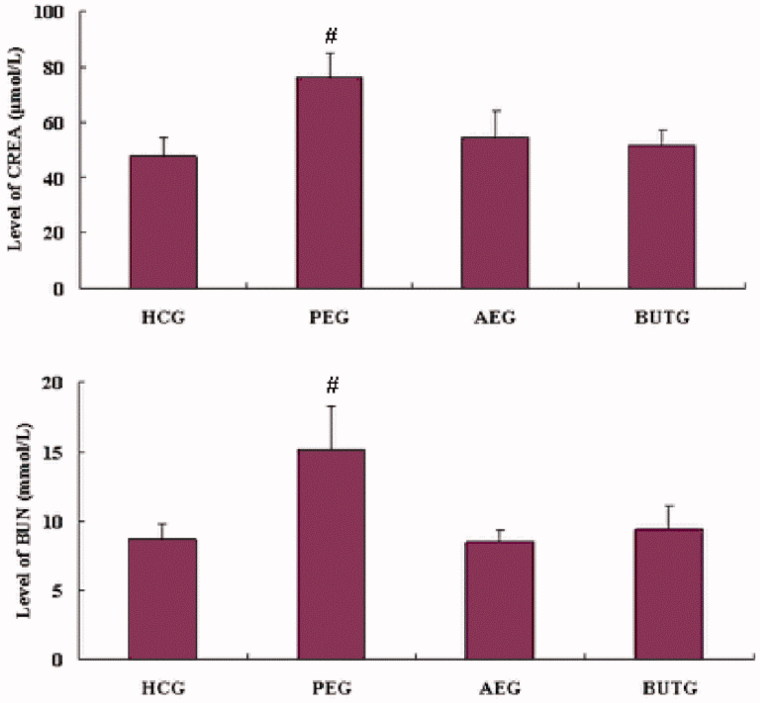
Clinical chemistry: the value of creatinine (CREA) and blood urea nitrogen (BUN) (*n* = 8). #Compared with HCG (*p* < 0.01).

The average values of CREA in PE, AE, BUT and control groups were 76.54 ± 9.52, 54.12 ± 10.34, 51.33 ± 5.19 and 48.23 ± 6.67 μmol/L, respectively. The average values of BUN in PE, AE, BUT and control groups were 15.25 ± 3.37, 8.32 ± 0.89, 9.22 ± 1.78 and 8.47 ± 1.33 mmol/L, respectively. All the results are presented in Figure 1. CREA is widely interpreted as a measure of the glomerular filtration rate (GFR) and is used as an index of renal function in clinical practice as well as BUN. The average values of CREA and BUN in PEG increased significantly (*p* < 0.01) in comparison with HCG, indicating that renal damage might happen. In the contrast, the values in other groups did not show difference from that of HCG.

### Histopathology

**Figure 2.**
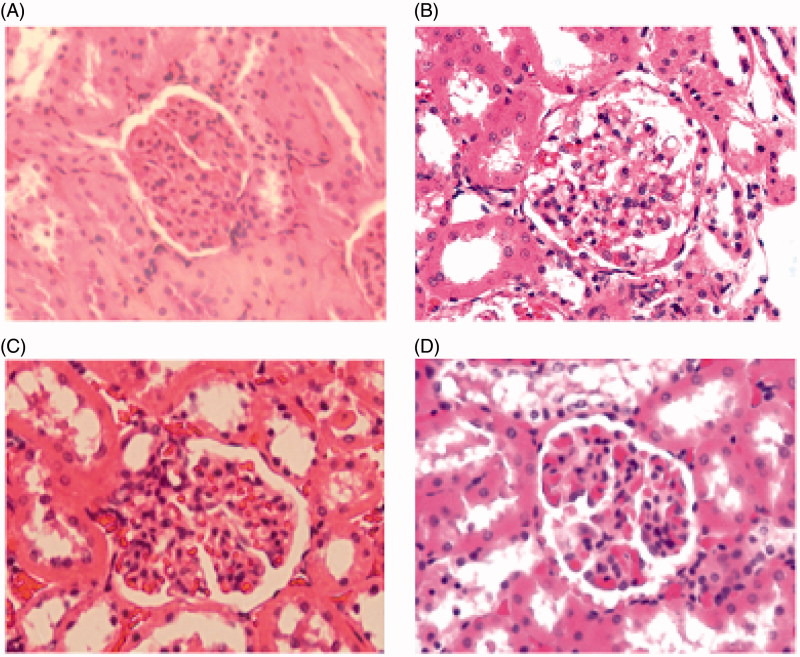
Histopathological photomicrographs of rat kidney sections (40×).

Histopathological findings after administration of different section extracts of EPR are summarized in Figure 2. As shown in Figure 2(A,C,D), the kidney section of the HCG, AEG and BUTG showed apparently normal structure in renal cortex and medulla, while the kidney section from PEG (Figure 2(B)) showed that glomeruli had cellular or fibrocellular crescents. Histopathological results confirmed the presence of substantial kidney damage after the administration of PE section of EPR in line with serum biochemical parameters, indicating that PE section should be responsible for the renal toxicity. To investigate the mechanism of renal damage and the constituents in PE section, metabolomics and identification study were carried out in the following experiments.

## Kidney metabolomics study

When renal injury happened, some metabolic pathways were disordered, leading to level changes of biological metabolites in the kidney. After PE section was screened as the nephrotoxicity section of EPR, the kidney samples of PEG were applied in the metabolomics study to find out the possible nephrotoxicity mechanism of EPR.

### Data analysis of metabolomics study

**Figure 3.**
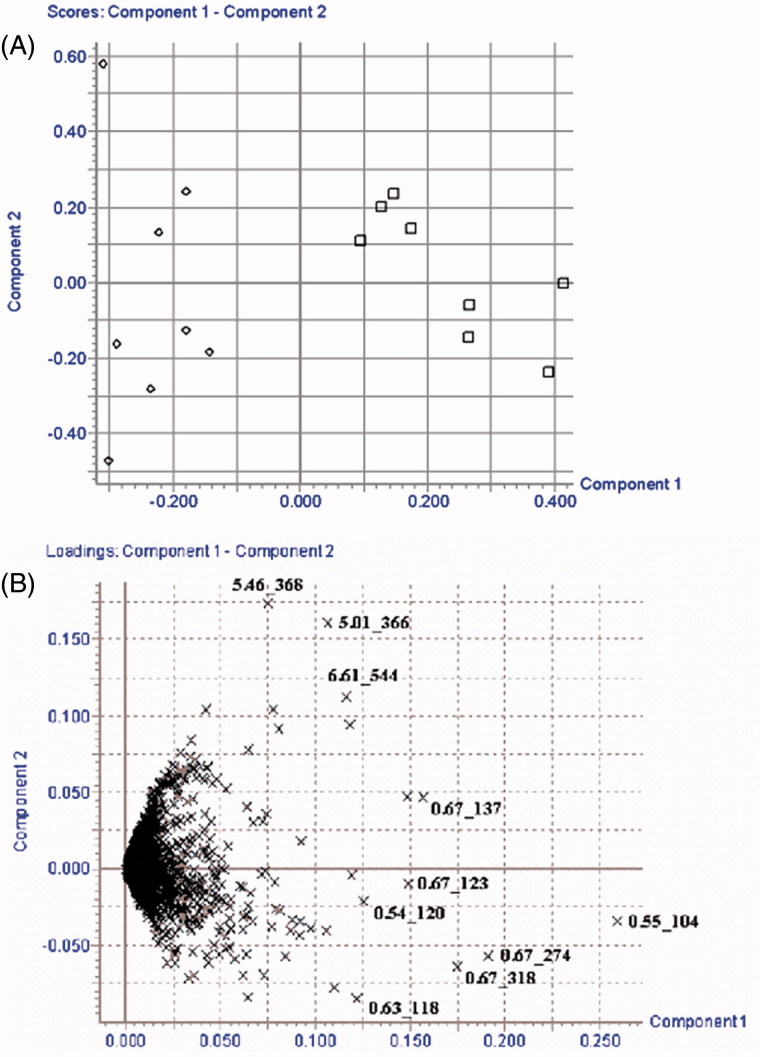
PCA score plot (A) and corresponding loading plot (B) of kidney samples in the time-point of 10th week post-dose for rats administrated with PE section of EPR. (□) HCG and (△) PEG.

Principal component analysis (PCA), a chemometric model which reduces matrix of data to lowest dimension of the most significant factors, was used to gain a comprehensive view of the metabolome for analysing the chromatographic data. The obtained PCA score plot from the processing of data is shown in Figure 3(A), which could be readily divided into two clusters: HCG and PEG. The samples of HCG tend to cluster in the left, while the PEG was located in the right. The clearly separation in Figure 3(A) indicated the physiological status of the rats in these two groups were quite different. Based on the phenomenon showed by the PCA score plot coupled with the serum biochemistry and histopathology results, the presence of substantial kidney injury after administration of PE section of EPR was confirmed.

### Biomarker identification of metabolomics study

**Figure 4.**
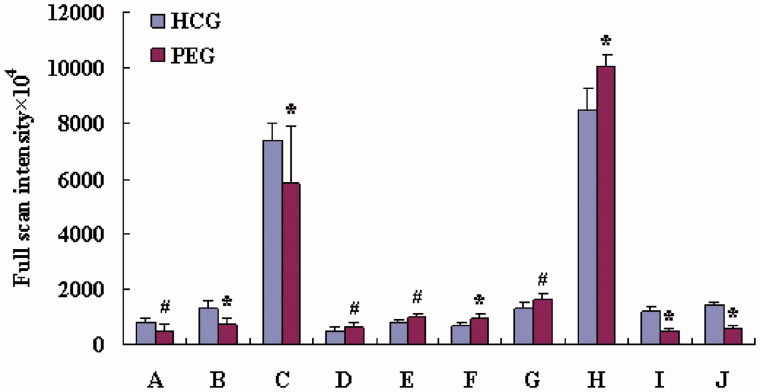
The comparison of biomarker intensity between HCG and PEG. (A) Hypoxanthine, (B) LPC C 20:4, (C) niacinamide, (D) phenylalanine, (E) C_16_ dihydrosphingosine, (F) C_18_ phytosphingosine, (G) *N*,*N*-dimethylglycine, (H) unknown, (I) unknown and (J) unknown. #Compared with HCG (*p* < 0.05) and *compared with HCG (*p* < 0.01).

In order to gain insight into the metabolic changes of the kidney injury induced by the PE section of EPR, the loading profile (Figure 3(B)) that visualizes the influences of variables was used for the selection of biomarkers. These metabolites were correlated to the distinction between normal and abnormal conditions. Judged by the distance from the origin, a series of ions which were found predominantly in the loading plot were chosen as biomarkers (Table 1). Moreover, the full scan intensity of potential biomarkers in HCG and PEG was also taken into consideration (Figure 4). In our study, the identifications of the biomarkers were done using the commercial available standards by comparing their MS/MS spectra and retention time.

**Table 1.** Potential kidney biomarkers of nephrotoxicity induced by EPR.

Retention time (min)	*m*/*z* (Da)	Scan mode	Quasi-molecular ion	Metabolite	Deviation ofnormal plasma
0.67	137	+	[M + H]^+^	Hypoxanthine	↓
6.61	544	+	[M + H]^+^	LPC C 20:4	↓
0.67	123	+	[M + H]^+^	Niacinamide	↓
0.54	120	+	[M + H–HCOOH]^+^	Phenylalanine	↑
0.67	274	+	[M + H]^+^	C_16_ dihydrosphingosine	↑
0.67	318	+	[M + H]^+^	C_18_ phytosphingosine	↑
0.55	104	+	[M + H]^+^	*N*,*N*-Dimethylglycine	↑
0.63	118	+	Unknown	Unknown	↑
5.01	366	+	Unknown	Unknown	↓
5.46	368	+	Unknown	Unknown	↓

### Biochemical interpretation

During the study period, the main biomarkers displayed in Table 1 were hypoxanthine, LPC, niacinamide, phenylalanine, *N,N*-dimethylglycine (DMG), C_16_ dihydrosphingosine, C_18_ phytosphingosine and three unknown biomarkers.

The kidney metabolite profile results indicated that the hypoxanthine level was significantly decreased in the PE-treated rats compared with the HCG rats. Hypoxanthine is a spontaneous deamination product of adenine, and the relationship between hypoxanthine and renal damage is due to the proteolytic conversion of xanthine dehydrogenase to xanthine oxidase (Zhao et al. 2013). What is more, the end product of hypoxanthine catabolism is uric acid which could deposit in renal tissue and form renal calculi. As hypoxanthine was decreased in this study, uric acid was likely to accumulate in kidney and produce renal damage.

LPC, a major component of oxidized-low density lipoproteins, modulated various pathobiological processes involved in vascular and glomerular diseases. Previous research reported that oxidative stress is related to renal damage (Rahman et al. 2003). LPC as an endogenous metabolite which possesses surface activity can ruin pericellular membrane of different tissues including kidney and liver, and further lead to intensity hepatic and kidney injury (Sekas et al. 1985; Joles et al. 1994). The change in LPC content may be a probable result of PE-induced kidney injury.

Niacinamide is a component of nicotinamide adenine dinucleotide (NAD), playing a very important role in the energy metabolism. The research had reported that niacinamide probably causes thrombocytopenia in renal dialysis patients (He et al. 2014). The relationship between niacinamide and renal failure might be both the disturbance of energy metabolic pathway and the change of thrombocyte levels.

Amino acids play important roles in our daily life. Phenylalanine and DMG are two essential amino acids in human body. Phenylalanine is mainly hydroxylated by phenylalanine hydroxylase to tyrosine. In our study, phenylalanine was markedly increased in the PE-treated group compared with the HCG group, indicating renal damage induced by PE section. DMG is a metabolite of homocysteine. Some studies have shown plasma DMG might accumulate in chronic renal failure and contribute to hyperhomocysteinaemia by inhibiting betaine homocysteine methyltransferase activity (McGregor et al. 2001). The disturbance of amino acids metabolism might be one of the nephrotoxicity mechanisms of EPR.

Sphingolipids are essential components of cell membranes, including dihydrosphingosine and phytosphingosine. Dihydrosphingosine can be transformed to phytosphingosine *in vivo* by C4-hydroxylase, which plays an important role in the anabolic metabolism and catabolism of sphingolipids (Riezman 2006). Some important metabolites occurring in sphingolipid metabolism, such as ceramide and sphingosine-1-phosphate, are produced by the action of different enzymes. In the present study, there was an obvious increase in dihydrosphingosine and phytosphingosine in the model group compared with the control group, which will result in a high level of ceramides being produced. It has been reported that, as an important intracellular secondary messenger, ceramide has a major effect on cell apoptosis, which may bring growth cytotoxic and inhibitory activities for kidney cell systems (Hannun et al. 1991).

The changes of selected and identified potential biomarkers suggested the involvement of some specific metabolic pathways, such as purine metabolism, phospholipids metabolism, energy metabolism, amino acid metabolism and sphingolipids metabolism. It may be of great importance for getting an insight into development of renal damage and revealing the metabolism of renal damage induced by PE section.

## Nephrotoxic constituents’ investigation

In order to confirm the toxicity material basis, a further study was performed to systematically characterize for multiple absorbed constituents in rat serum after oral administration of PE section of EPR by HPLC/Q-TOF-MS method.

### Analysis of PE section of EPR

**Figure 5.**
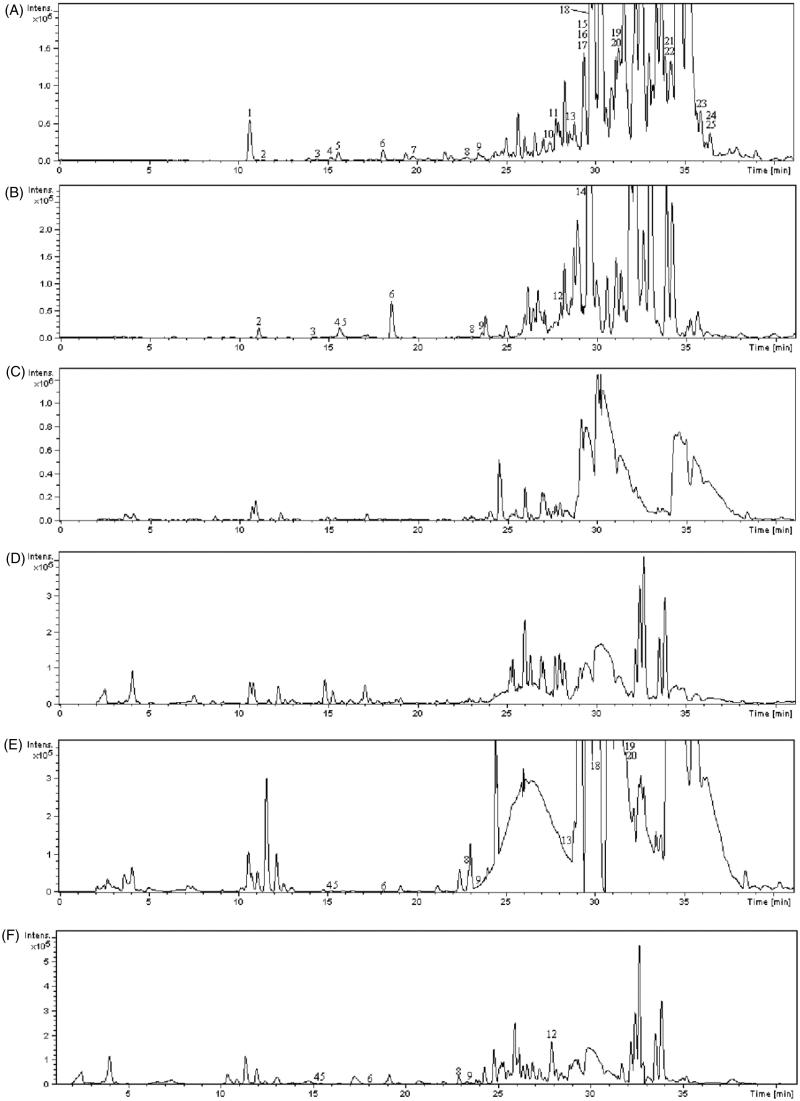
A set of chromatograms of (A) petroleum ether extract of EPR in positive ion mode, (B) petroleum ether extract of EPR in negative ion mode, (C) blank rat serum in positive ion mode, (D) blank rat serum in negative ion mode, (E) 1 h rat serum sample after oral administration of petroleum ether extract of EPR in positive ion mode and (F) 1 h rat serum sample after oral administration of petroleum ether section of EPR in negative ion mode.

**Figure 6.**
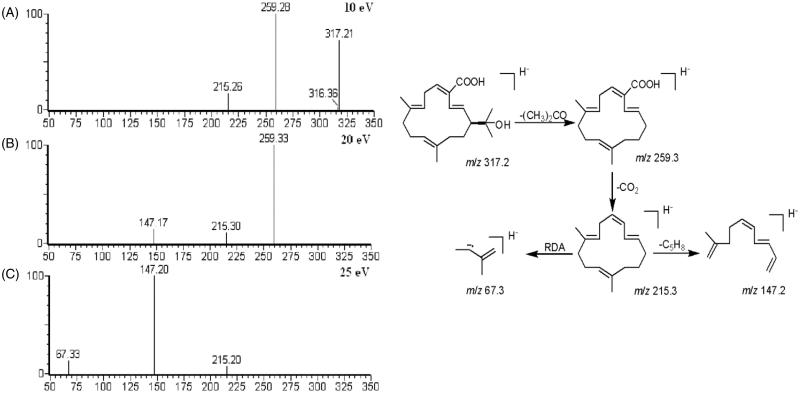
Product ion scan spectra in negative ion mode under different collision energies (10, 20 and 25 eV) and possible MS fragmentation mechanism of (–)-(1*S*)-15-hydroxy-18-carboxycembrene.

**Figure 7.**
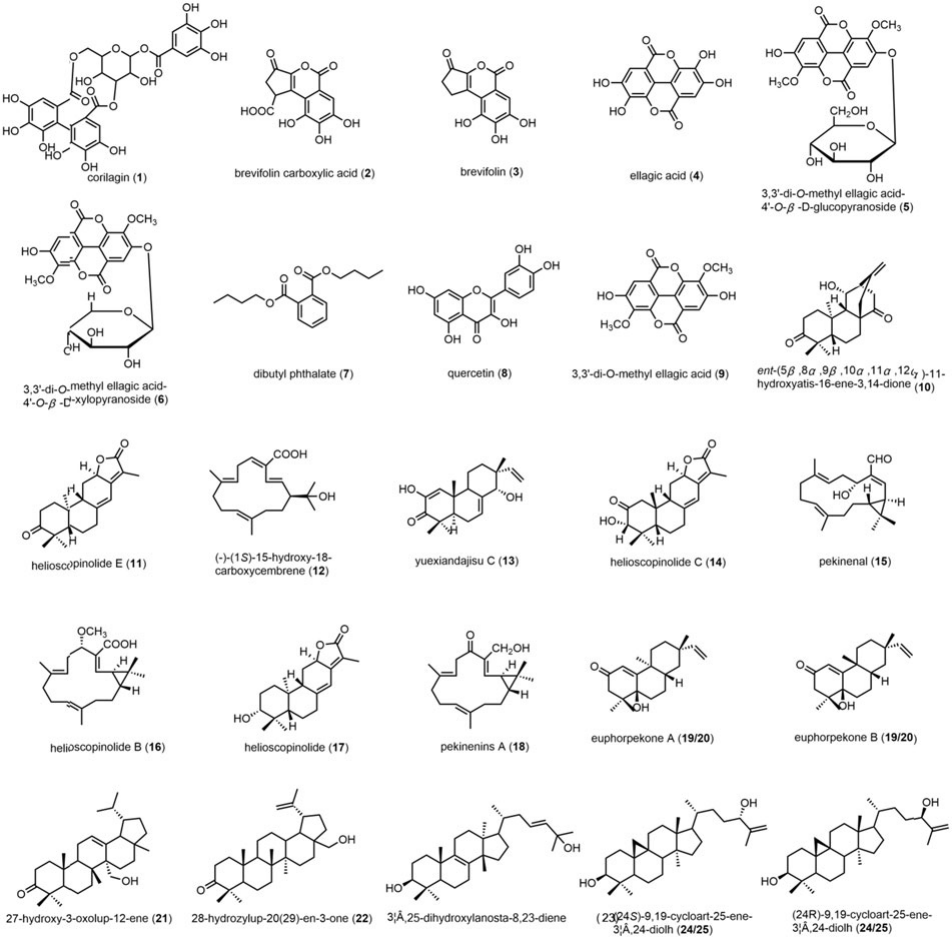
Chemical structures of compounds identified from PE section.

Base peak and extracted ion chromatograms of the PE section of EPR are shown in Figure 5(A,B). The constituents in the PE section of EPR were well separated by the HPLC/Q-TOF MS method and 25 compounds were identified, which were identified by comparison with the reference standards, utilizing Elemental Composition software to carefully investigate their MS spectra and comparing with the literature data. By comparing with the authentic compound, peak 12 was unambiguously identified as (–)-(1*S*)-15-hydroxy-18-carboxycembrene, taken as an example to illustrate the identification process. In the first-order mass spectra, peak 12 showed [M–H]^–^ at *m*/*z* 317.2 from ESI^–^ ion mode. Under different collision energies (10, 20 and 30 eV), peak 12 was fragmented to acquired necessary ion information. The MS/MS figure contains high abundance fragment ion at *m*/*z* 259.3, 215.3, 147.2 and 67.3, which represented the fragments [M–H-2CH_3_-CO]^–^, [M–H-2CH_3_-CO-CO_2_]^–^, [M–H-2CH_3_-CO-CO_2_-C_5_H_8_]^–^ and CH_2_–CH**–**CH–CH**–**CH_2_, respectively (Figure 6). Finally, on the basis of the retention time, mass-to-charge ratio and product ions by comparing with the authentic compound, peak 12 was identified as (–)-(1*S*)-15-hydroxy-18-carboxycembrene. Other compounds were also identified by the same way. The MS data containing MS spectra information are listed in Table 2 and their chemical structures are shown in Figure 7. These results provided reliable information for confirming the relative molecular masses and structures of the chemical constituents.

**Table 2.** MS data (*m*/*z*) of the compounds of PE section of EPR and in rat serum after oral administration of PE section of EPR.

Peak no.	*t*_R_ (min)	Quasi-molecularion	Formula	Measuredmass (*m*/*z*)	Calculatedmass (*m*/*z*)	mDa	Compound
1	10.8	[M + Na]^+^	C_27_H_22_Na_1_O_18_	657.0709	657.0698	–1.04	Corilagin
2	11.3	[M + H]^+^	C_13_H_9_O_8_	293.0292	293.0292	–0.01	Brevifolin carboxylic acid
		[M–H]^–^	C_13_H_7_O_8_	291.0204	291.0146	–5.74	
3	14.1	[M + H]^+^	C_12_H_9_O_6_	249.0393	249.0394	0.07	Brevifolin
		[M–H]^–^	C_12_H_7_O_6_	247.0273	247.0248	–2.52	
4^a^	15.2	[M + H]^+^	C_14_H_7_O_8_	303.0162	303.0135	–2.68	Ellagic acid
		[M–H]^–^	C_14_H_5_O_8_	301.0008	300.9990	–1.81	
5^a^	15.5	[M + H]^+^	C_22_H_21_O_13_	493.0920	493.0977	5.66	3,3′-Di-*O*-methyl ellagic acid-4′-*O*-*β*-d-glucopyranoside
		[M–H]^–^	C_22_H_19_O_13_	491.0861	491.0831	–2.99	
6^a^	18.1	[M + H]^+^	C_21_H_19_O_12_	463.0903	463.0871	–3.16	3,3′-Di-*O*-methyl ellagic acid-4′-*O*-*β*-d-xylopyranoside
		[M–H]^–^	C_21_H_17_O_12_	461.0730	461.0725	–0.47	
7	19.8	[M + H]^+^	C_16_H_23_O_4_	279.1581	279.1591	0.95	Dibutyl phthalate
8^a^	22.9	[M + H]^+^	C_15_H_11_O_7_	303.0500	303.0499	–0.10	Quercetin
		[M–H]^–^	C_15_H_9_O_7_	301.0340	301.0354	1.39	
9^a^	23.4	[M + H]^+^	C_16_H_11_O_8_	331.0448	331.0448	0.04	3,3′-Di-*O*-methyl ellagic acid
		[M–H]^–^	C_16_H_9_O_8_	329.0304	329.0303	–0.08	
10	27.7	[M + H]^+^	C_20_H_29_O_3_	317.2091	317.2111	2.02	ent-(5*β*,8*α*,9*β*,10*α*,11*α*,12*α*)-11-Hydroxyatis-16-ene-3,14-dione
11	27.9	[M + H]^+^	C_20_H_27_O_3_	315.1936	315.1955	1.86	Helioscopinolide E
12^a^	28.0	[M–H]^–^	C_20_H_29_O_3_	317.2124	317.2122	–0.16	(–)-(1*S*)-15-Hydroxy-18-carboxycembrene
13^a^	28.5	[M + H]^+^	C_20_H_29_O_3_	317.2078	317.2111	3.33	Yuexiandajisu C
14	29.2	[M–H]^–^	C_20_H_25_O_4_	329.1784	329.1758	–2.59	Helioscopinolide C
15	29.7	[M + Na]^+^	C_20_H_30_Na_1_O_2_	325.2127	325.2138	1.13	Pekinenal
16	29.7	[M + H]^+^	C_20_H_29_O_3_	317.2069	317.2111	4.22	Helioscopinolide B
17	29.8	[M + H]^+^	C_20_H_29_O_3_	317.2063	317.2111	4.83	Helioscopinolide
18^a^	30.2	[M + Na]^+^	C_20_H_30_Na_1_O_2_	325.2120	325.2138	1.84	Pekinenins A
19^a^	31.7	[M + H]^+^	C_20_H_31_O_2_	303.2274	303.2319	4.47	Euphorpekone A
20^a^	31.7	[M + H]^+^	C_20_H_31_O_2_	303.2274	303.2319	4.47	Euphorpekone B
21	34.5	[M + H]^+^	C_30_H_49_O_2_	441.3643	441.3727	8.44	27-Hydroxy-3-oxolup-12-ene
22	34.7	[M + H]^+^	C_30_H_49_O_2_	441.3642	441.3727	8.55	28-Hydrozylup-20(29)-en-3-one
23	35.9	[M + H–H_2_O]^+^	C_30_H_49_O	425.3657	425.3778	12.05	3*β*,25-Dihydroxylanosta-8,23-diene
24	36.3	[M + H–H_2_O]^+^	C_30_H_49_O	425.3659	425.3778	11.91	(24S)-9,19-Cycloart-25-ene-3*β*,24-diolh
25	36.3	[M + H–H_2_O]^+^	C_30_H_49_O	425.3659	425.3778	11.91	(24R)-9,19-Cycloart-25-ene-3*β*,24-diolh

a Compounds in rat serum after oral administration of PE section of EPR.

### Analysis of serum samples of PE section

To clarify the venenosus constituents responsible for the nephrotoxicity, it is necessary to know the chemical constituent profile *in vivo*. Therefore, rat serum sample after oral administration of PE section of EPR was analysed by the same HPLC/Q-TOF method. Base peak and extracted ion chromatograms of blank serum sample and rat serum sample after administration PE section of EPR are shown in Figure 5(C–F). The MS data of the ESI (±)-MS spectra are listed in Table 2 and their chemical structures are shown in Figure 7. There were 10 peaks displayed in the profiles of PE section of EPR, whereas there were no equivalent peaks in the profile of the blank serum or blank solvent. Thus, these compounds were defined as prototype components, and identified as diterpenes and ellagitannins.

According to the retention times and mass spectra with those of authentic compounds, peaks 4, 5, 6, 8, 9, 12 and 13 were designated as ellagic acid, 3,3′-di-*O*-methyl ellagic acid-4'-*O*-β-d-glucopyranoside, 3,3′-di-*O*-methyl ellagic acid-4′-*O*-β-d-xylopyrano-side, quercetin, 3,3′-di-*O*-methyl ellagic acid, (–)-(1*S*)-15-hydroxy-18-carboxy-cembrene, yuexiandajisu C, respectively.

As is well known, only compounds absorbed into the blood have the probability to be toxic constituents; therefore, the 10 compounds identified in rat serum might be the potentially nephrotoxicity ingredients of EPR to induce nephrotoxicity. It is necessary to research the toxicological activities of these 10 compounds by other *in vitro* or *in vivo* experiments in the future work.

## Conclusions

The nephrotoxicity of PE, AE and BUT section of EPR ethanol extract was investigated to find out the nephrotoxic section of EPR with the combination of serum biochemical parameters and histopathology results. According to the results, PE section was thought to be the nephrotoxic section of EPR and used for the further study. A good separation was seen between rats treated with PE and healthy rats in the PCA score plot of kidney metabolites by the first two components, and 10 potential biomarkers were screened out and identified by using metabonomic method combined with multivariate data analysis. These results showed abnormal metabolism of purine, phospholipids, energy, amino acid and sphingolipids in rats treated with PE section. Then, an HPLC/Q-TOF method was developed and used to search for the toxic ingredient in PE section and in rat serum after oral administration of PE section. As a result, 25 compounds in PE section and 10 prototype components in serum were successfully separated and identified by comparing retention times and MS spectra with those of authentic compounds and literature data. This identification and structural elucidation of these constituents would be also helpful to reveal the toxicodynamic profile of EPR, which will facilitate its clinical usage. The strategy to find out potential nephrotoxicity ingredients will give a better and further understanding of toxic effects and mechanism research of TCM in the future.
